# Changes in axial length, central cornea thickness, and anterior chamber depth after rhegmatogenous retinal detachment repair

**DOI:** 10.1186/s12886-016-0296-z

**Published:** 2016-07-25

**Authors:** Chunmei Huang, Tonghe Zhang, Jian Liu, Qiang Ji, Ruili Tan

**Affiliations:** Department of Retina and Vitreous, The Second People’s Hospital of Jinan, 148# Jingyi Road, Jinan, 250001 People’s Republic of China

**Keywords:** Anterior chamber depth, Axial length, Central cornea thickness, Optical coherence tomography, Retinal detachment

## Abstract

**Background:**

This study was designed to measure changes in anterior chamber depth (ACD), central cornea thickness (CCT), and axial length (AL) after scleral buckle (SB) surgery or pars plana vitrectomy (PPV) for the repair of rhegmatogenous retinal detachment (RD).

**Methods:**

We prospectively reviewed the records of 245 eyes of 245 patients scheduled to undergo SB surgery and 238 eyes of 238 patients scheduled to undergo PPV. ACD, CCT, and AL were measured by spectral-domain optical coherence tomography (SD-OCT) and biometry, before surgery as well as 6 and 12 months postoperatively.

**Results:**

For both SB and PPV surgeries, ACD was observed to decrease significantly postoperatively, with this trend continuing throughout the follow-up period (*p* < 0.005). CCT showed no significant difference after PPV or SB surgery. AL increased significantly after SB surgery but not PPV.

**Conclusion:**

Our results show that SB surgery altered the shape of the eye by changing both ACD and AL. PPV resulted in altered ACD. These findings should elucidate the changes to be expected after SB and PPV surgeries.

## Background

A rhegmatogenous retinal detachment (RD) involves pathologic separation of the neural retina from the pigment epithelium because of a hole or break in the retina. Rhegmatogenous RD leads to the loss of visual function and requires prompt surgical therapy. RDs can be managed by pneumatic retinopexy, scleral buckling (SB) or vitreoretinal surgery. SB involves the use of an encircling element and was introduced in 1957 by Schepens et al. These external techniques may not only lead to changes in axial length but may also cause a reduction in anterior chamber depth (ACD) [1–4].

SB and vitrectomy can induce secondary glaucoma and changes in refractive error, most commonly, myopic shift. SB surgery changes the shape of the eye, altering the degree of corneal curvature as well as AL [5–10]. Anterior segment-optical coherence tomography (AS-OCT; Visante, Carl Zeiss Meditec AG 07745, Jena, Germany) is widely employed for clinical examination of the anterior segment. AS-OCT is a non-contact imaging method that provides high-resolution cross-sectional images of the anterior chamber angle and cornea as well as measurements of ACD. Several studies have established the technology’s high reproducibility and repeatability, which are equal to or even better than other techniques currently used to measure ACD [11]. The IOL Master is now widely used to obtain noninvasive, accurate AL measurements. The validity and repeatability of these measurements have been established [12].

Using the AS-OCT and IOL Master, we evaluated changes in ACD, central corneal thickness (CCT), and axial length (AL) after SB and pars plana vitrectomy (PPV) surgeries. We compare our findings for both surgeries. These results should elucidate the changes to be expected after SB and PPV surgeries. This study investigates the postoperative complications for RRD patients and provides information to guide surgery selection. The results have profound clinical significance.

## Methods

This prospective study included 483 eyes of 483 patients with primary rhegmatogenous RD seen at the Department of Retina and Vitreous at the Second People’s Hospital of Jinan, Shandong province, China, during the period from September 2010 to December 2013. The inclusion criteria for the study were rhegmatogenous RD without age-related macular degeneration, cataract, vitreous hemorrhage, anterior proliferative vitreoretinopathy, choroidal detachment, macular holes, trauma, macular edema, intraocular inflammation, glaucoma, or retinal vascular occlusive diseases. Eyes with preexisting corneal disease were also excluded. Patients with systemic diseases, such as diabetes or hypertension, were excluded. Eyes that had undergone cataract, glaucoma, or refractive surgery were excluded from the study. Ultimately, SB (Group A) and PPV (Group B) were successfully performed in 245 and 238 eyes, respectively. Informed consent to participate in the study according to the tenets of the Declaration of Helsinki was obtained from all participants. The study was approved by the ethical committee of the Second People’s Hospital of Jinan, Shandong province, China.

In Group A, retinal cryocoagulation was performed around the retinal breaks, which had been localized with precision. All patients underwent standard SB surgery. The segmental element was placed circumferentially without an encircling band. The segmental element was fixed 12–14 mm posterior to the limbus with 5–0 silk sutures. No gas or air was used in any of the SB surgeries. In Group B, all eyes underwent a standard 23-gauge 3-port PPV, laser photocoagulation, fluid-air exchange, and silicone oil or C3F8 tamponade at the end of the surgery. All vitrectomies were performed without SB. All patients underwent pre- and postoperative best-corrected logMAR visual acuity (VA) testing, slit-lamp biomicroscopy, autorefractometry, intraocular pressure (IOP) assessments, and dilated fundus examinations. AS-OCT was used to measure CCT and ACD. ACD was measured from the corneal endothelium to the anterior pole of the crystalline lens. The sectional plane is horizontal (0–180°). CCT was measured from the epithelium to the endothelium. Post-operative AS-OCT and IOL Master measurements were obtained on undilated eyes with the patient in the seated position. A single researcher (JL) calculated ACD and CCT using AS-OCT software. AS-OCT and IOL Master measurements were obtained with the patient undilated, in the seated position. AS-OCT and IOL Master measurements were also obtained 6 and 12 months after surgery. The data are presented as mean ± standard deviation (SD). Statistical analyses included independent *t*-test and repeated measure ANOVA. The level of statistical significance was set at *p* < 0.005.

## Results

High intraocular pressure occurred in group A (5 eyes) and group B (13eyes) patients within 2 weeks postoperative. Redetachment happened in 12 cases (12eyes) of group B and the 12 patients underwent second surgery. There were no other surgical complications among group A and group B. The average age of group A patients was 36.6 ± 17.1 years (range: 16–68 years); 119 were women (48.6 %), and 126 were men (51.4 %). The average age of Group B patients was 44.7 ± 12.8 years (range: 21–71 years); 118 were women (49.6 %), and 120 were men (50.4 %). The characteristics of patients see Table 1.

**Table 1:** characteristics of patients in the scleral buckling and vitrectomy groups

Variable	Scleral buckling	Vitrectomy
	(*n* = 245)	(*n* = 238)
Age, mean ± SD	36.6 ± 17.1	44.7 ± 12.8
(range)	16–68	21–71
Sex		
Males	126	120
Females	119	118
IOP (mmHg), mean ± SD	Before 15.7 ± 2.4	16.4 ± 1.9
	6 m 16.7 ± 1.6	17.6 ± 1.2
	12 m 18.0 ± 1.1	17.9 ± 2.3
refractive errors (D)	before 0.8 ± 0.6	1.0 ± 0.4
	6 m 2.7 ± 0.5	1.3 ± 0.2
	12 m 2.4 ± 0.7	1.5 ± 0.1
PVR		
None	112	94
PVR A	94	69
PVR B	39	75
Macular involvement	80	165
C3f8 tamponade (eyes)	0	97
Silicone oil tamponade (eyes)	0	141

*Abbreviations*: *IOP* intraocular pressure, *Before* before surgery, *6 month* 6 months after surgery, *12 month* 12 month after surgery

**Fig. 1:**
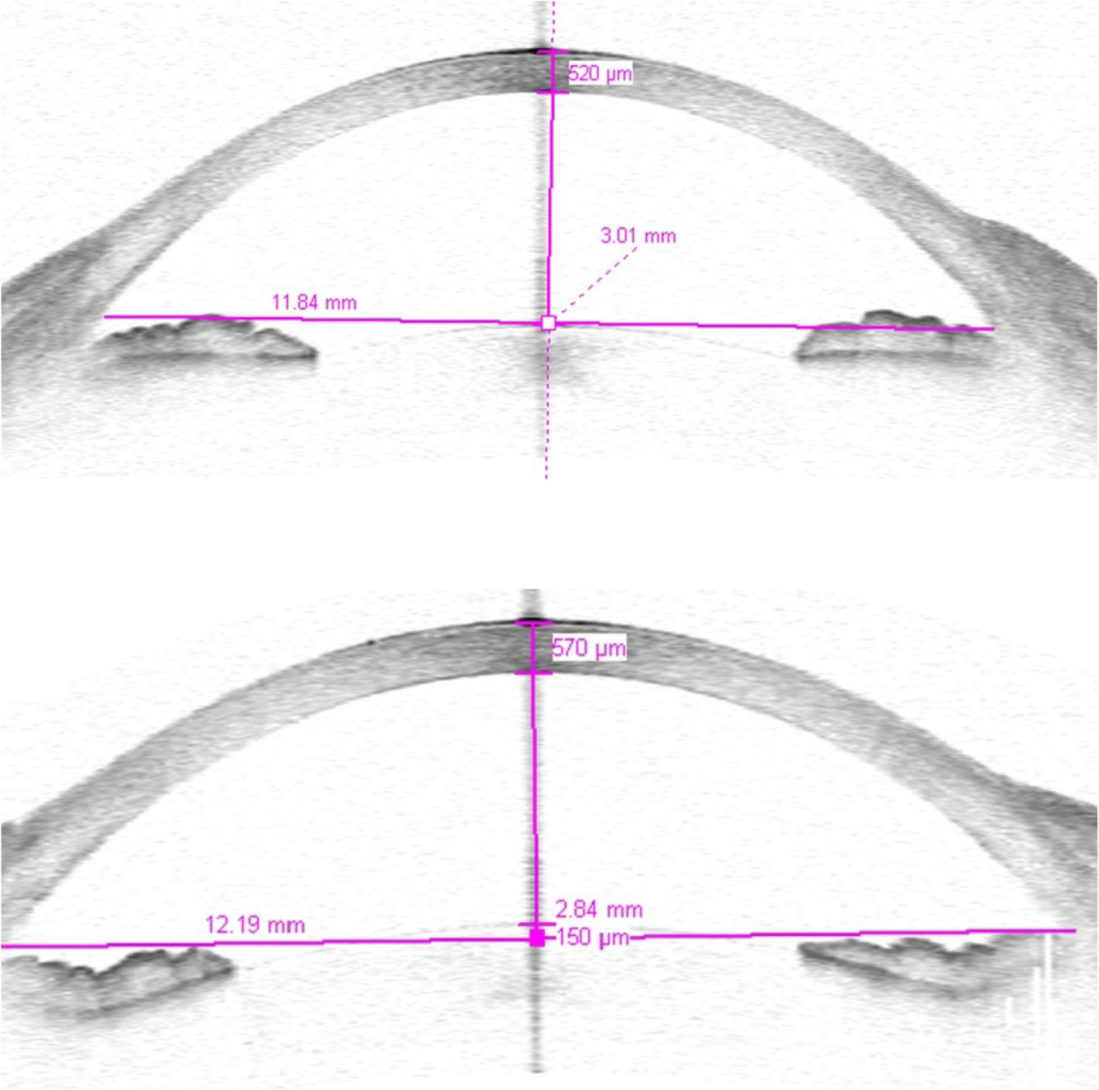
ACD was measured from the corneal endothelium to the anterior pole of the crystalline lens. The sectional plane is horizontal (0–180°). CCT was measured from the epithelium to the endothelium. **1** Rhegmatogenous RD patient, 64 years old, treated by vitrectomy. Preoperative ACD, 3.01 mm; CCT, 520 mm. **2** 6 months Postoperative ACD, 2.84 mm; CCT, 570 mm

**Fig. 2:**
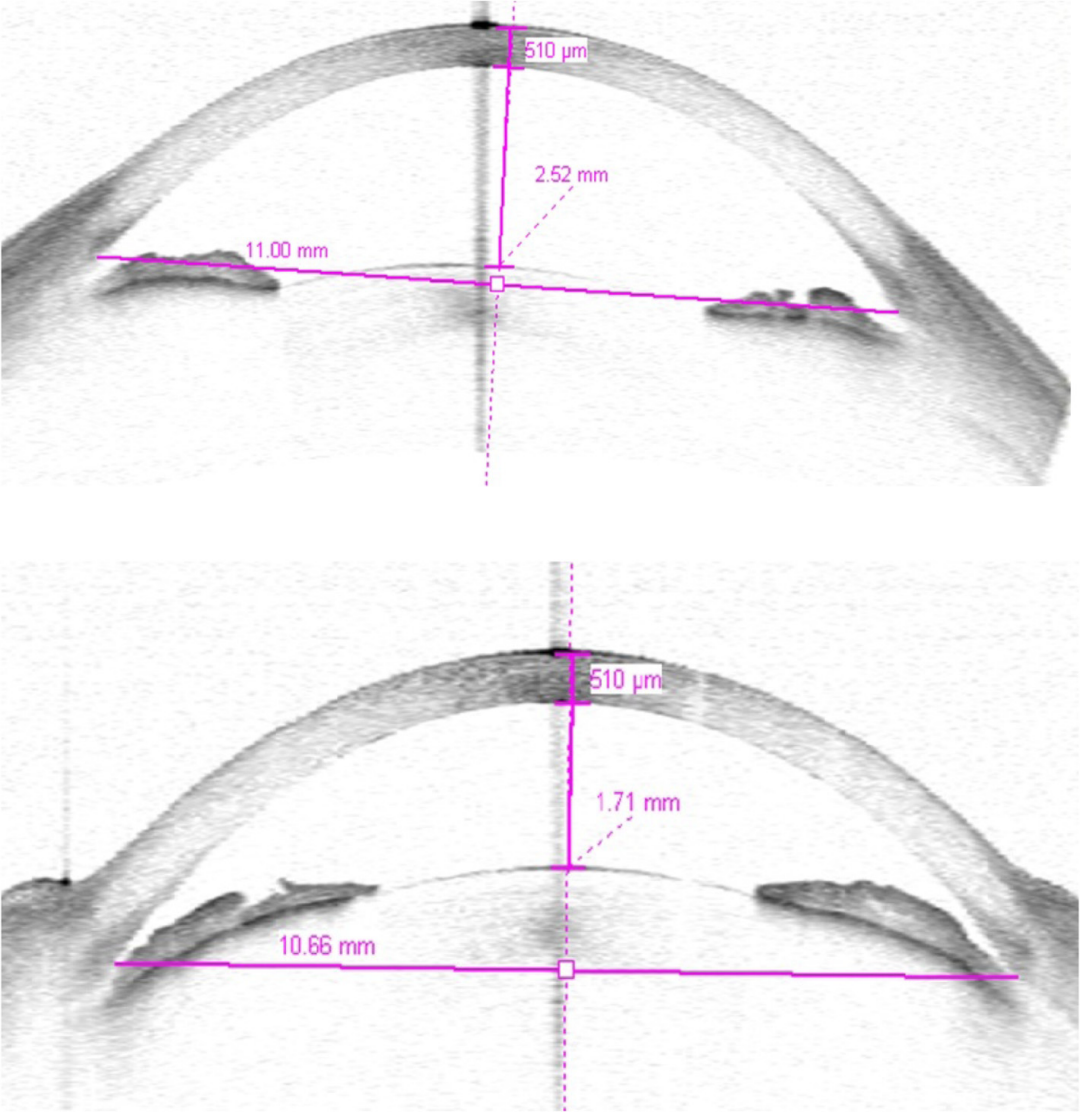
**1**: Rhegmatogenous RD patient, 48 years old, treated by scleral buckle. Preoperative ACD, 2.52 mm; CCT, 510 mm. **2**: 6 months Postoperative ACD, 1.71 mm; CCT, 510 mm

In Group A, mean ACD was 3.20 ± 0.46 mm preoperatively, 3.01 ± 0.34 mm after 6 months, and 3.03 ± 0.25 after 12 months (*p* < 0.005; Table 2). In Group B, mean ACD was 3.07 ± 0.28 mm preoperatively, 2.97 ± 0.31 mm after 6 months, and 3.02 ± 0.16 mm after 12 months (Table 3). The difference between preoperative and postoperative ACD measurements was significant in both groups (*p* < 0.005; Figs. 1 and 2).

**Table 2:** Preoperative and postoperative AS-OCT and IOL-MASTER findings of Group A (*n* = 245)

	Preoperative	6 month postoperatively	12 month postoperatively
CCT (mm)	560 ± 43	555 ± 36	548 ± 15
ACD (mm)	3.20 ± 0.46	3.01 ± 0.34	3.03 ± 0.25
AL (mm)	23.97 ± 2.31	25.13 ± 3.11	25.25 ± 2.07

**Table 3:** Preoperative and postoperative AS-OCT and IOL-MASTER findings of Group B (*n* = 238)

	Preoperative	6 month postoperative	12 month postoperative
CCT (mm)	554 ± 29	563 ± 28	550 ± 19
ACD (mm)	3.07 ± 0.28	2.97 ± 0.31	3.02 ± 0.16
AL (mm)	24.15 ± 4.27	24.78 ± 3.65	24.37 ± 2.90

Mean preoperative CCT was 560 ± 43 mm in Group A (Table 1) and 554 ± 29 mm in Group B (Table 2). Mean CCT as measured 6 month postoperatively was 555 ± 36 mm in Group A (Table 1) and 563 ± 28 mm in Group B (Table 2). 12 months after the operation, mean CCT in Groups A and B was 548 ± 15 mm (Table 2) and 550 ± 19 mm (Table 3), respectively. In Group A and B the postoperative measurements were not significantly different from those obtained preoperatively (*p* > 0.005; Figs. 1 and 2).

In Group A, mean AL increased from 23.97 ± 2.31 mm preoperatively to 25.13 ± 3.11 mm after 6 months and to 25.25 ± 2.07 mm after 12 months (*p* < 0.005; Table 2). In Group B, AL was similar before (24.15 ± 4.27 mm) and after surgery (24.78 ± 3.65 mm and 24.37 ± 2.90 mm) (*p* > 0.005; Table 3). In Group B, The silicone oil was aspirated at 3 months postoperatively and the C3F8 was completely absorbed at 6 weeks ~ 8 weeks postoperatively. The ACD, AL and CCT were not significantly different for C3F8 or silicone oil tamponade eyes at 6 or 12 month follow-up.

## Discussion

The measurements used for our study were obtained by AS-OCT, a non-contact anterior-segment imaging method. Lavanya et al [13] and Nemeth et al [11] showed that ACD measurements obtained by AS-OCT were significantly deeper than those obtained with the IOL Master or US immersion A-scan (UltraScan Imaging System, Alcon Laboratories, Fort Worth, TX). ACD values caused by indentation of the cornea and shallowing of the anterior chamber with the probe tip of the US device. The US for ACD measurement with direct corneal contact, which may result in inaccurate off-axis measurement, the differences in probe tip handling and the different settings of US velocity also cause error. All AS-OCT measurements are performed on axis with the moveable built-in fixation target. AS-OCT measurement is more accurate. In the present study, we used AS-OCT to measure CCT and ACD. Our findings are consistent with previous findings from other studies [14, 15].

Herein, we reported the changes in ACD, AL and CCT following vitrectomy or SB for rhegmatogenous RD. We found that SB as well as PPV caused significant changes in ACD. Previous studies have found that changes in lens thickness and reductions in ACD after scleral buckle surgery have altered the patient’s refractive error [16]. Potential reasons for the observed changes include the following factors. Mechanical pressure: a scleral buckle can displace the lens, or increase intraocular pressure enough to increase lens convexity [16]. Trauma during surgery could stimulate contraction of the ciliary body muscle, resulting in increased lens thickness. Abnormally low intraocular pressure prior to surgery may have resulted in falsely high measurements of ACD. Scleral buckle surgery may impede blood flow and homeostasis postoperatively [17]. Burton et al. found increased lens thickness for at least 6 weeks after SB surgery [1]. Notably, methodological differences among studies may also account for this discrepancy in ACD measurements.

In the past, RDs were repaired using hard silicone explants or scleral implants [1, 4]. We used scleral explants for SB surgery to avoid the need for scleral dissection; this may have contributed to the observed reduction in ACD. Ultrasound biomicroscopic studies after SB surgery demonstrated that ciliary effusion shifted the iris-lens diaphragm anteriorly, which resulted in a shallower anterior chamber [18]. The SB may have impeded uveal or retinochoroidal circulation, triggering ciliary body edema [19]. These factors, in combination with compression of the vitreous by the SB, may have decreased ACD. In the PPV group, the patient’s face-down position or the silicone oil tamponade may have shifted the lens-iris diaphragm forward. In addition, puncturing the pars plana may have induced ciliary body edema.

A number of studies have reported that SB surgery alters the shape of the eye by changing both corneal curvature and AL [5–10]. Many of these studies used AS-OCT to measure the associated changes in CCT. Fiore and Newton4 evaluated CCT after SB surgery with a pachymeter mounted on a slit-lamp. All of the eyes that had undergone surgery exhibited corneal thickening that had subsided by 2 months postoperatively. Additionally, it was previously reported that the CCT increased significantly after vitrectomy. The thickness recovered to the preoperative levels at 1 month after the surgery [20]. It was also noted that the degree of the increase in the corneal thickness was affected by the degree of invasiveness of the vitrectomy. Therefore, corneal thickness measurements may be useful for assessing the extent of the surgical stress. In our study, no significant changes occurred in CCT both group A and group B at 6 and 12 months postoperatively. In our study, The cornea thickness of both PPV and SB were not significantly changed 6 months and 12 months postoperatively.

This study found that after scleral buckling surgery and vitrectomy surgery, at long-term follow-up, 6 months and 12 month, the CCT showed no signifant difference than preoperative, ACD deepened than preoperative level at 6 months and 12 month follow up. The axial length increased after scleral buckling. This trend continued until 12 months after surgery. Refractive changes after surgery were consistent with the axial length changes. The myopic shift in the SB group at 6 months and 12 months postoperatively with respect to the preoperative values was statistically significant (*p* < 0.005). Smiddy et al. [21] showed a myopic shift of −3.95 D at 6 months after scleral buckling. As for astigmatic changes, different studies showed up to a 1.5D increase within the first postoperative month that decreased to below 0.5D after 3 months [3,5,21]. However, the analysis of this aspect was not included in our study.

The advantages of our study were the long follow-up time, the big sample size and the full application of SD-OCT in the retinal detachment surgerys. To a certain degree, it guide doctors to choose the operation mode and deal with the complications of surgery. Under these circumstance such as Patients of RD with shallow anterior chamber angle, decreased ACD, and a tendency of primary angle closure glaucoma, the doctor would take some necessary measures to avoid the occurrence of complications.

## Conclusions

The results of the present study suggest that SB surgery altered the shape of the eye by changing both ACD and AL. PPV resulted in altered ACD. These findings should elucidate the changes to be expected after SB and PPV surgeries. It guide doctors to choose the operation mode and deal with the complications of surgery.

## Abbreviations

ACD, Anterior chamber depth; AL, Axial length; CCT, Central cornea thickness; IOP, Intraocular pressure; PPV, Pars plana vitrectomy; RRD, Rhegmatogenous retinal detachment; VA, Visual acuity
